# Relationships between Iron Status and Selected Physical Fitness Components of South African Adolescents: The PAHL-Study

**DOI:** 10.3390/children11060659

**Published:** 2024-05-28

**Authors:** Makama Andries Monyeki, Tamrin Veldsman, Ben Coetzee, Martinique Sparks, Sarah Johanna Moss, Cindy Pienaar, Mariette Swanepoel, Linda Malan, Herculina Salome Kruger

**Affiliations:** 1 Physical Activity, Sport, and Recreation Research Focus Area (PhASRec), Faculty of Health Sciences, North-West University, Potchefstroom 2520, South Africa; tamrin.veldsman@nwu.ac.za (T.V.); ben.coetzee@nwu.ac.za (B.C.); martinique.sparks@nwu.ac.za (M.S.); hanlie.moss@nwu.ac.za (S.J.M.); cindyp@dut.ac.za (C.P.); mariette.swanepoel@nwu.ac.za (M.S.); 2 School of Health and Medical Sciences, University of Southern Queensland, Ipswich, QLD 4305, Australia; 3 Centre for Health Research, University of Southern Queensland, Ipswich, QLD 4305, Australia; 4 Department of Sport Studies, Durban University of Technology, Kwa-Zulu Natal 4000, South Africa; 5 Centre of Excellence for Nutrition, Faculty of Health Sciences, North-West University, Potchefstroom 2520, South Africa; linda.malan@nwu.ac.za (L.M.); salome.kruger@nwu.ac.za (H.S.K.); 6 Medical Research Council Research Unit for Hypertension and Cardiovascular Disease, Faculty of Health Sciences, North-West University, Potchefstroom 2520, South Africa

**Keywords:** cardiorespiratory fitness, muscular strength, iron status, haemoglobin, BMI, South African adolescents

## Abstract

Poor iron status is detrimental to physical and cognitive performance in adolescents. Due to the limited studies investigating the association between iron status and physical fitness components in adolescents from low- and middle-income countries, we aimed to determine the association of iron status with selected physical fitness components in South African adolescents. A cross-sectional study design, including 178 adolescents (102 girls and 76 boys) from the Physical Activity and Health Longitudinal Study (PAHLS), was followed. Height and weight were measured to calculate the body mass index (BMI). Subsequently, WHO BMI-for-age-specific categorised body fatness. Cardiorespiratory fitness was determined with a 20-m shuttle run test (${\overset{\text{˙}}{V}O}_{2\max}$), and lower-body explosive power by the standing broad jump (SBJ). Fasting haemoglobin (Hb) and ferritin were analysed from blood samples. Correlation analyses determine the association between iron status, explosive power and cardiorespiratory fitness. Of the 178 participants, 18.5% (*n* = 33) had low Hb, and 14% (*n* = 25) iron deficiency without anaemia. Significant positive correlations were found between the selected physical fitness components, ferritin, and Hb. In boys, a positive association was found between Hb and SBJ (*r* = 0.30, *p* = 0.006), whilst in girls, positive associations were found between ferritin (*r* = 0.25, *p* = 0.04) and SBJ, and Hb with both SBJ (*r* = 0.21, *p* = 0.03) and ${\overset{\text{˙}}{V}O}_{2\max}$ (*r* = 0.32, *p* = 0.001). Hb concentration remained associated with ${\overset{\text{˙}}{V}O}_{2\max}$ and SBJ in girls after adjustment for age, whilst in boys, Hb concentration was associated with SBJ. Higher iron status in South African adolescents is associated with higher lower-limb explosive power and cardiorespiratory fitness. We suggest monitoring of haematological parameters, and interventions to improve the iron status of South African adolescents.

## 1. Introduction

Physical fitness and adequate micronutrient intake, particularly iron, are important factors in young people’s current and future health outcomes (e.g., growth and physical performance) [1,2,3]. Physical fitness encompasses the execution of activities demanding aerobic endurance, strength, and flexibility, predominantly influenced by inherited genetic traits, consistent physical activity, and a measure of health [4,5]. Adolescents’ cardiorespiratory endurance, an indicator of physical fitness, has been on the decline [6] due to many possibilities, including decreased physical activity and an increased prevalence of overweight/obesity [7]. Lack of physical activity influences the physical, psychological, social, and cognitive health of adolescents [7]. Cardiorespiratory endurance, which challenges the circulatory system, may also be affected by iron deficiency and anaemia considering the role of iron. Iron in the human body consists of active iron [haemoglobin (Hb) (65%), myoglobin (10%), and enzymes (5%)]; depot iron [ferritin (20%)], and transport iron [transferrin (0.1–0.2%)] [8]. Anaemia, therefore, is defined as Hb below 12 g/dL in girls aged 12–15 years and <13 g/dL in girls 15–18 years of age [9,10].

Haemoglobin is essential for transporting oxygen from the respiratory system to the muscular system of the entire body; therefore, Hb deficiency will decrease oxygen transport for normal daily functions [11]. Additionally, various researchers have indicated the importance of iron status and Hb concentrations on cardiovascular endurance and muscular strength [1,8,12,13,14]. Since all forms of iron deficiency affect physical fitness and performance in adolescence [8], significant and strong positive correlations were observed between the ${\overset{\text{˙}}{V}O}_{2\max}$ and Hb mass in children of all ages [15,16,17]. Schmidt and Prommer [16] also found that a change in 1 g/kg of Hb mass leads to an increase of 4 mL/kg/min in ${\overset{\text{˙}}{V}O}_{2\max}$. Additionally, relative plasma and blood volume mass (g/kg) are strongly associated with VO_2peak_ [15,17]. Furthermore, significant positive relationships between Hb mass (g/kg) and endurance performance (*r* = 0.627, *p* = 0.035) were found in adolescents performing endurance sports [16]. In the Healthy Lifestyle in Europe by Nutrition in Adolescence-Cross-sectional Study (HELENA-CSS), a positive association was also found between Hb concentrations, muscle capacity (assessed by SBJ), and heart tenacity (cardiorespiratory endurance, determined by ${\overset{\text{˙}}{V}O}_{2\max}$) in boys and adolescents [14].

Globally, one-quarter of adolescents are suffering from iron deficiency anaemia, and 19% are reported to be from Africa [18]. Africa has the highest prevalence of anaemia in children and adolescents (5–15 years). Over 60% of sub-Saharan African children reportedly present with anaemia [10]. Another study on South African children reported a prevalence of 11–20% for iron deficiency among adolescents aged 15 to 18 years [19]. In Southeast Asia, more than 50% of girls aged 12–15 years are diagnosed with anaemia, while the prevalence among boys remains [20,21]. However, in Nepal, the percentage was comparably lower, with 20.6% of girls and 10.9% of boys diagnosed as anaemic, compared to a higher prevalence of 23% among the South African youth population [22,23].

Adolescence is a significant window period, characterised by physical and physiological changes, warranting research and intervention to circumvent the susceptible development of risk associated with obesity, inactivity, and anaemia [24]. Adolescence is also a period during which the risk of iron deficiency increases due to the high iron requirements of the adolescent growth spurt [25]. However, female adolescents are at a greater risk of iron deficiency [26] due to menarche, rapid growth, and nutritional limitations, placing a greater demand on the body’s iron stores [1,27]. Given the importance of iron as an essential nutrient in the energy metabolism, immune function, and cognitive development of children and adolescents, it raises various health concerns because iron deficiency and anaemia are prevalent in developing countries, such as South Africa [10,19,28]. However, despite the importance of iron status in adolescents’ development, research on the relationship between iron status and selected physical fitness indicators in adolescents is limited. The limited data is especially prominent in Africa, of which South Africa is not an exception. In the South African context, malnutrition is a major risk factor influencing the development of adolescents physically and mentally in under-resourced areas. Given these shortcomings, we sought to determine the relationships between iron status and selected physical fitness indicators of South African adolescents. In our study, we hypothesised that a significant positive relationship exists between the iron status, cardiorespiratory fitness as predicted ${\overset{\text{˙}}{V}O}_{2\max}$ (based on the 20-m shuttle run test), and the SBJ for the lower-body explosive power of South African adolescents.

## 2. Materials and Methods

### 2.1. Design

This cross-sectional study is part of the Physical Activity and Health Longitudinal (PAHL) study—“a five-year multidisciplinary longitudinal study on physical activity and health” (2010–2014) [29]. The overall aim of the PAHL study was to determine adolescents’ health-related physical fitness (HRPF) components, body composition, and physical activity levels. However, in this study, only data collected in 2012 with a one-time measurement point for the outcome variables of blood samples for iron indicators and selected physical fitness of the same participants in PAHLS was analysed. The PAHL study was conducted in the JB Marks Local Municipality (previously known as Tlokwe Local Municipality) within the Dr Kenneth Kaunda District Municipality of the North West Province of South Africa. The municipality is located between 26°43′0″ S and 27°6′0″ E and has a longitude of 27, 1000 (276′0.000″ E). The JB Marks Local Municipality has a population density of 34/km^2^ and a total area coverage of 6.398 km^2^ (STATSSA) [30]. 

### 2.2. Study Sample

Participants consisted of 178 healthy (75 boys, 103 girls), 13–16-year-old adolescents (mean ± SD; 14.82 ± 0.71 years) who met the following inclusion criteria: healthy boys and girls, grades 8 and 9, who signed parental informed consent, and participant assent to participate in the study. The Road to Health Clinic Card confirmed each adolescent’s birth date. Demographic information included sex, race, and location (i.e., town or township) [29].

### 2.3. Data Collection

#### 2.3.1. Body Composition

Height and weight were measured by level two accredited Kinanthropometrists using the protocol of the International Standard of Advancement of Kinanthropometry (ISAK) [31]. The height was measured to the nearest 0.1 cm using a Seca 213 stadiometer (Birmingham, UK) while participants stood barefoot and upright with their heads in the Frankfort plane. The weight was measured with a consistent electronic scale (Seca 813 digital) to the nearest 0.1 kg, with the participants wearing minimal clothing. The body mass index (BMI) was calculated as body mass/stature^2^ (kg/m^2^). Subsequently, the corresponding WHO age-specific BMI for children was used to determine the following categories: overweight, normal weight, and underweight/thinness [32,33].

#### 2.3.2. Blood Analysis for Iron Status Assessment

A professional nurse collected venous blood from the vena cephalica of the adolescents who fasted for 12 h before blood sampling. Ethylenediaminetetra-acetic acid (EDTA) whole blood was used to measure Hb in g/dL with an AcT 5diff Cap Pierce Hematology Analyzer (Beckam Coulter, Miami, FL, USA) with three-level controls provided by the manufacturer. The serum was prepared by storing the tubes for about 30 min to coagulate and then centrifuging for 15 min at 2000 g. The serum was divided into aliquots and stored at −80 °C. Serum was used to analyse iron, ferritin, transferrin, and % transferrin saturation with enzyme-linked immunosorbent assays (Ramco Laboratories, Inc., Stafford, Texas) at an accredited laboratory (Ampath Laboratories, Pretoria, South Africa). All analyses’ quality control had a coefficient of variance (CV) of <10%.

Anaemia was defined as Hb < 12 g/dL in girls adolescents between 12 to 15 years of age, or <13 g/dL in boys and adolescent girls older than 15 years. Iron deficiency was defined as serum ferritin (SF) below 15 µg/L, while iron deficiency anaemia was defined as both Hb below 12 g/dL (girls) or <13 g/dL (boys and girls > 15 years) and SF < 15 µg/L [34].

### 2.4. Selected Physical Fitness Components

The 20-m shuttle run was conducted to predict cardiorespiratory endurance as ${\overset{\text{˙}}{V}O}_{2\max}$ and the SBJ as lower-body explosive power using standardised test protocols [35,36].

#### 2.4.1. Cardiorespiratory Endurance

Predicted ${\overset{\text{˙}}{V}O}_{2\max}$ was assessed by the validated 20-m shuttle run test (20-m SRT) as an indicator of adolescents’ cardiorespiratory endurance [37]. A 15-min warm-up was performed before the start of the 20-m SRT. The warm-up consisted of aerobic exercises with shorter, high intensity running exercises and dynamic stretches. Participants ran the 20-m SRT barefoot on a flat, clearly marked 20-m space on the outdoor non-slip surface of the different school grounds. They had to run back and forth on the 20-m track, pacing themselves to arrive at the end of the 20-m on the beep signal emitted from a commercially available pre-recorded compact disk (20-m Shuttle Run Test CD) [36]. Marking lines at either end of the 20-m had to be touched with one foot as the signal sounded. The 20-m SRT had to be run at a progressively faster running speed, increasing every minute of the test to indicate a change in test level. Verbal encouragement was given to motivate the participants to perform the maximally. The test ended if participants voluntarily dropped out or did not reach either end mark of the 20-m distance in two successive shuttles when the beep signal was emitted. The levels of the last shuttle completed before dropping out were recorded. Verbal encouragement was given to motivate the participants to perform maximally. The recorded 20-m SRT levels were converted to a predicted maximal oxygen consumption (${\overset{\text{˙}}{V}O}_{2\max}$) in millilitres of oxygen used in one minute per kilogram of body weight (mL/kg/min: ${\overset{\text{˙}}{V}O}_{2\max}$, mL/kg/min), and the final score was cross-checked against the reference level and shuttle number of the instruction booklet that accompanied the 20-m SRT CD [36].

Validity studies of the 20-m SRT demonstrated significant and non-significant correlations of *r* = 0.77–0.87 (*p* < 0.001) and *r* = 0.72 (*p* > 0.05) between the directly measured ${\overset{\text{˙}}{V}O}_{2\max}$ and 20-m SRT estimated ${\overset{\text{˙}}{V}O}_{2\max}$ in children and adolescents [38,39].

#### 2.4.2. Lower Extremity Explosive Power

Lower extremity explosive power was measured with the SBJ from the EUROFIT [35] protocol. The distance jumped in centimetres (cm) indicated the lower extremity explosive power. The SBJ is a reliable (r = 0.89–0.9) and valid test to determine participants’ peak anaerobic power output [40]. The SBJ distance was recorded as the distance from the zero line to the two-legged jump distance of the heel that was closest to the zero line. Participants took off from both feet and jumped forward as far as possible from a still-standing position. Each participant was allowed two trials, and the better of the two trials was used in the final analysis. 

### 2.5. Ethical Considerations

This study was conducted following the Declaration of Helsinki guidelines, and all procedures involving human subjects were approved by the Health Research Ethics Committee in 2010 of the North-West University where the research was conducted (Ethics no: NWU-0058-01-A1). Permission was also received from the North West Province Department of Health and Social Welfare Research Committee and the Department of Education. The informed consent of the parents/guardians of the adolescents was obtained in writing, and oral consent was recorded and verified. In addition to parental/guardian consent, the adolescents also provided assent to take part in this study.

### 2.6. Statistical Analysis 

The Statistical Package for the Social Sciences (SPSS) version 28 (SPSS, Inc., Chicago, IL, USA) was used for data analysis. The distribution of data was checked for normality using the Kolmogorov-Smirnov test and QQ plots. Differences between boys and girls were evaluated using Mann-Whitney *U* tests for non-normally distributed variables and independent *t*-tests for variables with a normal distribution. The Kruskal-Wallis test was used to compare the iron and anaemia status. Spearman’s correlation coefficients were used to assess the relationships between iron status and HRPF (SBJ and ${\overset{\text{˙}}{V}O}_{2\max}$). Partial correlations adjusted for age were also calculated, separately for boys and girls. Correlation coefficients were interpreted following Cohen [41] cut-points as follows: *r* = 0.1–0.29 is considered a weak correlation; *r* = 0.3–0.49 a moderate correlation; and *r* = 0.5–1.0 a strong correlation. The level of statistical significance was set at *p ≤* 0.05.

## 3. Results

Of the total number (*n*) of 178 adolescents, 18.5% (*n* = 33) had low Hb levels/anaemia, 14.0% (*n* = 25) had low ferritin/iron deficiency, and 27% of the girls had low Hb compared to 7% of boys (Table 1). More girls suffer from iron deficiency without anaemia (21%) compared to boys (4%). Seventy-one percent of adolescents presented with a normal weight; 5.1% were underweight, 18.5% were overweight, and 4.5% were obese. More girls than boys were overweight (23.3 vs. 12%). In the sample, no participant presented with a risk of iron overload (SF > 15 µg/L girls, SF > 20 µg/L boys).

The results from the iron status and selected physical fitness measurements showed that the participants with iron deficiency without anaemia and low Hb performed significantly (*p* < 0.001) poorly in the SBJ and ${\overset{\text{˙}}{V}O}_{2\max}$, especially for girls compared to the boys (Table 2).

Table 3 shows the comparisons between boys and girls. The boys were significantly taller and heavier than the girls. The blood analysis indicated that boys had significantly higher mean values of iron, serum ferritin, transferrin saturation (%), and Hb compared to girls. The boys obtained significantly better values/measures than the girls for the SBJ and ${\overset{\text{˙}}{V}O}_{2\max}$ (*p* < 0.001).

Body mass index was significantly and negatively associated with ${\overset{\text{˙}}{V}O}_{2\max}$, and SBJ in the total group (Table 4). In the whole group, Hb was moderately and positively associated with both outcomes in girls but only with SBJ in boys. Ferritin was moderately and positively associated with ${\overset{\text{˙}}{V}O}_{2\max}$, and SBJ in the whole group and in girls.

Further analysis (Table 5) of the observed significant associations was done separately for boys and girls adjusted for age, showing that Hb concentration remained moderately associated with cardiorespiratory fitness and SBJ in boys, while in girls, Hb concentration was associated with SBJ.

## 4. Discussion

This present study aimed to determine the association between iron status and selected physical fitness components of South African adolescents and found that Hb concentrations were positively associated with cardiorespiratory endurance in girls and lower extremity power in both boys and girls. Serum ferritin was significantly and positively associated with SBJ in girls. 

In our study, 21% of girls and 4% of boys had iron deficiency without anaemia, which was in the same range as the 23% reported in the South African youth population [19,23]. Girls in the present study mostly presented with 27% low Hb and 21% iron deficiency without anaemia, and this may be an affirmation of the known theory that female adolescents are at a greater risk of iron deficiency [26]. The high prevalence of iron deficiency among girls observed in our present study is similar to the reported values in Nepalese girls [22]. However, the prevalence of iron deficiency in boys in our study is lower compared to Nepalese boys [22]. The reason for the differences in the results can be explained by the sample size differences between the studies. The observed high prevalence of iron deficiency combined with 23% combined overweight and obesity, demonstrates the double burden of malnutrition in low- and middle-income countries [42], where iron deficiency, as well as overweight and obesity, occur in adolescent girls [25]. In an Ethiopian study, one in six adolescents was anaemic [43], compared to one in five in our study. In the Ethiopian study, the predictors of anaemia were family size, sex, fathers’ education status, and body mass index [43]. Possible reasons for the variance in the prevalence of iron deficiency may include educational background and the lower level of nutritional education in low- and middle-income countries compared to high-income countries. Also, the different cut-points used in studies may be a reason; for example, in a study of 193 elite young German athletes from 24 different sports low iron stores were present in 31% of male and 57% of female athletes. They used serum ferritin < 35 μg/L as the cut-off, and with a more stringent criterion (serum ferritin < 12 μg/L) in the same group of athletes, only 4% of boys and 7% of girls were deemed to have depleted iron stores [44]. Despite physical and physiological changes occurring during adolescence in both boys and girls, girls are three times more likely to develop anaemia than boys [43]. This might be partly explained by the fact that as the demand for nutrients increases, the risk of nutrient deficiency increases [45]. Adolescents are exposed to the risk of iron deficiency due to a combination of iron loss during the menstrual cycle in girls and rapid growth (peak height velocity/growth spurt) in boys [46]. High-intensity physical activity is also a contributor to iron deficiency in adolescents [47].

The results of this current study show that 18.5% of the participants are overweight, 4.5% obese, and 5.1% are underweight. More girls than boys were overweight (23.3 vs. 12.0%), while 5.5% of boys and 4.9% of girls were underweight. The observed prevalence of underweight, overweight, and obesity in the current study is similar to the first South African Youth Risk Behaviour (YRB) survey, whereby 17.2% were overweight and 9.0% were underweight, (boys 15.6% and girls 3.9%) [48]. Of concern, though, are the established links between excess overweight and obesity and anaemia and low iron status in adolescents [20], hence the negative effect of anaemia on cognitive development [10,19,28], and physical fitness and performance [9], which warrant intervention in curbing or managing of the condition so as not to persist into adulthood.

Iron deficiency without anaemia has been shown to significantly impair both athletes’ and non-athletes ability to optimize the performance of physical fitness tests [47]. It was evident from the results that participants with iron deficiency without anaemia and low Hb performed significantly (*p* < 0.001) poorly in the SBJ and ${\overset{\text{˙}}{V}O}_{2\max}$ assessments. Also, boys performed better than girls in the SBJ and ${\overset{\text{˙}}{V}O}_{2\max}$ physical fitness components. The findings are consistent with research published on adolescents, where boys outperformed girls in both lower limb muscular strength (jumping) and cardiorespiratory endurance (${\overset{\text{˙}}{V}O}_{2\max}$) [49]. The participants in the study are not athletes, and therefore, the observed significantly better performance in SBJ and ${\overset{\text{˙}}{V}O}_{2\max}$ physical fitness tests by boys than girls may partly be explained by the dominant culture of regular engagement in physical activity and muscular development factors among boys. BMI is statistically significant and inversely related to total ${\overset{\text{˙}}{V}O}_{2\max}$ and SBJ in the total group. However, when analysis was performed by sex, BMI was not significantly correlated with SBJ in boys. The observed non-significant correlation coefficient may be attributed to, firstly, the similar percentage (6.7%) of underweight and overweight (5.3%) among boys, as both underweight and overweight individuals are unable to perform physical fitness tests that require a high energy flux over a short period, such as SBJ that require force to move their bodies forward [49,50], and secondly, to the high SBJ mean value (185.84 ± 25.62 cm). A sedentary lifestyle leads to excessive body weight or body fat increases, which consequently increases the body’s load, causing higher insulin secretion, and an even higher level of sedentary behaviour [51]. The observed average predicted ${\overset{\text{˙}}{V}O}_{2\max}$ in our study is 40.13 ± 7.32 mL/kg/min for boys and 28.40 ± 5.30 mL/kg/min for girls which are well below the average predicted ${\overset{\text{˙}}{V}O}_{2\max}$ of 47.7 mL/kg/min from international data of girls aged 12 to 14 years and 49 to 52.1 mL/kg/min for boys aged 13 and 14 years [14,52,53,54,55]. As such, the low cardiorespiratory endurance value in our study and the overweight status of the participating girls may point to a risk for the development of cardiovascular disease in adolescents [1,8,12,13,14,17]. Some researchers reported that an increase in obesity, increased sedentary time, decreased levels of moderate-to-vigorous physical activity, and social and economic changes may contribute to low and unhealthy cardiorespiratory endurance [56,57]. These results highlight the need for intervention to especially improve the anthropometric and cardiorespiratory risk profiles of the study population. 

BMI is used as a measurement to classify nutritional status and as an indicator of good health in adolescents and adults alike, as physically active people have a lower BMI [58]. Furthermore, some studies have shown that overweight and obese adolescents are less likely to participate in sports activities and tend to be less active than normal-weight adolescents [59,60]. In addition, lower physical activity increases the risk of cardiovascular disease in overweight and obese children, resulting in a reduction of cardiorespiratory endurance in children [61]. 

In the current study, iron status markers have a positive and significant relationship with SBJ in the total group of participants. Iron is essential for synthesising ATP, the fundamental energy currency of cells, which influences the explosive power required for a successful jump [62]. Also, previous studies showed that adolescent boys and girls have higher measured levels of oxidative enzymes than adults [63,64]. Because children and adolescents rely more on oxidative mechanisms and/or the oxygen-dependent resynthesis of creatine phosphate in the mitochondria, it could be hypothesized that the aerobic system is predominant, especially in adolescents of this age group [65,66]. Children’s increased reliance on myoglobin-rich, oxidative fibres may also impact these interactions [67], making Hb’s oxygen-carrying capacity important, especially during cardiorespiratory-related activities and tests such as the ${\overset{\text{˙}}{V}O}_{2\max}$ test. 

The findings of the study should be interpreted in light of the limitations identified. Not all targeted participants in the PAHL study were assessed. Some adolescents withdrew from the study because they feared the needles used in blood sampling, while others reported cultural or religious beliefs, resulting in a relatively small sample size. The study’s cross-sectional nature may also be a limitation because it could not establish causality, and therefore longitudinal or intervention studies are needed to determine the causality effect in the observed relationships. The small sample size may preclude researchers’ generalization of the results to adolescents in the North West province or South Africa. The lack of dietary information and evaluation of gross motor skills in the study are limitations of the study, as we were unable to indicate if the iron status was due to limited diet-related iron intake. Despite the noted limitations, we believe that the inclusion of blood samples for Hb and the selected physical fitness tests rather than relying on physical activity questionnaires allowed researchers to directly assess the iron status and associated physical fitness of adolescents is the strength of the study. 

## 5. Conclusions

The study concluded that there is a positive relationship between iron status and selected physical fitness indicators, and a relatively high prevalence of iron deficiency among adolescents living in the Tlokwe local municipality of the North West Province, South Africa. The high prevalence of iron deficiency and its association with low functional performance of the SBJ physical fitness test calls for nutrition interventions combined with physical activity interventions to address iron deficiencies, thus improving muscle strength to promote future physical activity and health outcomes.

## Figures and Tables

**Table 1:** Classification of iron and anthropometric status of the participants for the total group and according to sex.

		Total Group (*n* = 178)			Boys (*n* = 75)			Girls (*n* = 103)		
Blood Samples	Normal Reference Value [34]	Median	Low (%)	Normal (%)	Median	Low (*n*, %)	Normal (*n*, %)	Median	Low (*n*, %)	Normal (*n*, %)
Hb	>12.0 g/dL	14 (8.1, 17.7)	33 (18.5)	145 (81.5)	15.20 (12.3, 17.7)	5 (7)	70 (93)	13.70 (8.1, 16.4)	28 (27)	75 (73)
SF	15–150 µg/L	41 (3.0, 149)	25 (14.0)	153 (86)	52 (11.0, 149)	3 (4)	72 (96)	30 (3, 96)	22 (21)	81 (79)
**WHO BMI Z categories**										
Underweight, *n* (%)		9 (5.1)			4 (5.3)			5 (4.9)		
Normal weight, *n* (%)		128 (71.9)			58 (77.3)			70 (68)		
Overweight, *n* (%)		33 (18.5)			9 (12)			24 (23.3)		
Obese, *n* (%)		8 (4.5%)			4 (5.3)			4 (3.9)		

WHO, 2023; Hb deficiency = Hb < 12 g/dL in 12–15 years old adolescent girls & < 13 g/dL < 13 in 15 to 18 yrs old boys; SF = serum ferritin.

**Table 2:** Mean and standard deviations (SD) for the selected physical fitness components and iron status for the total group, for boys and girls.

Physical Fitness Component				Total Group (*n* = 177)		Boys (*n* = 75)		Girls (*n* = 103)
	Iron Variable	Categorical Groups	*n*	Mean ± SD	*N*	Mean ± SD	*N*	Mean ± SD
**SBJ** (cm)	SF	Iron Deficiency	25	147.32 ± 22.93	3	175.67 ± 20.98	22	143.45 ± 20.69 *
		Normal SF	152	168.28 ± 28.58	72	186.26 ± 25.83	81	152.09 ± 20.03 *
	Hb	Anaemia	33	28.54 ± 6.42	5	156.00 ± 11.66	28	148.82 ± 18.44 *
		Normal Hb	144	34.11 ± 8.56	70	187.97 ± 25.04	74	150.76 ± 21.17 *
${\overset{\text{˙}}{V}O}_{2\max}$ (mL/kg/min)	SF	Iron Deficiency	25	28.54 ± 6.42	3	41.83 ± 2.84	22	26.73 ± 4.20 *
		Normal SF	152	34.11 ± 8.56	72	40.06 ± 7.45	81	28.82 ± 5.470 *
	Hb	Anaemia	33	29.10 ± 6.61	5	37.31 ± 8.16	28	27.64 ± 5.22 *
		Normal Hb	144	34.29 ± 8.61	70	40.33 ± 7.28	75	28.65 ± 5.30 *

SBJ = standing broad jump; ${\overset{\text{˙}}{V}O}_{2\max}$ = maximal oxygen consumption; mL/kg/min = milliliters of oxygen used in one minute per kilogram of body weight; SF = serum ferritin; Hb = haemoglobin, SD = standard deviation; * *p* < 0.001 for the categorical groups.

**Table 3:** Descriptive statistics for the total group and by gender.

	Boys Mean ± SD	Girls Mean ± SD	*p*-Value
Age (years)	14.85 ± 0.67	14.80 ± 0.74	0.68
Stature (cm)	165.79 ± 10.04	158.02 ± 6.83	<0.001
Body weight (kg)	57.81 ± 14.64	53.24 ± 11.67	0.02
BMI (kg/m^2^)	20.80 ± 3.95	21.24 ± 3.98	0.47
SBJ (cm)	185.84 ± 25.62	149.91 ± 20.24	<0.001
${\overset{\text{˙}}{V}O}_{2\max}$ (mL/kg/min)	40.13 ± 7.32	28.40 ± 5.30	<0.001
Serum Iron (mmol/L)	17.46 ± 6.70	13.14 ± 5.66	<0.001
Transferrin (mg/dL)	3.06 ± 0.44	3.18 ± 0.45	0.068
Serum Ferritin (µg/L)	59.69 ± 32.73	35.43 ± 23.72	<0.001
Transferrin Saturation (%)	23.47 ± 9.88	17.20 ± 8.21	<0.001
Hb (g/dL)	15.16 ± 1.25	13.51 ± 1.50	<0.001

BMI = body mass index; Kg = kilogram; cm = centimeter; SBJ = standing broad jump; SD = standard deviation; ${\overset{\text{˙}}{V}O}_{2\max}$ = maximal oxygen consumption; mL/kg/min = milliliters of oxygen used in one minute per kilogram of body weight; mmol/L = millimoles per litre; mg/dL = milligrams (mg) per deciliter (dL); μg/L = micrograms per liter; g/dL = grams per deciliter; % = percentage; Hb = hemoglobin; statistical significance set at *p* ≤ 0.05.

**Table 4:** Correlation coefficient (*r*) between selected physical fitness components and iron status for the total participants and separately for boys and girls.

		Total		Boys		Girls	
		${\overset{\text{˙}}{V}O}_{2\max}$	SBJ	${\overset{\text{˙}}{V}O}_{2\max}$	SBJ	${\overset{\text{˙}}{V}O}_{2\max}$	SBJ
Serum Iron (mmol/L)	*r*	0.24 **	0.33 **	−0.02	0.22	0.08	0.13
	*p*	0.001	<0.001	0.88	0.06	0.45	0.21
Serum Ferritin (µg/L)	*r*	0.27 **	0.34 **	−0.12	0.03	0.16	0.25 *
	*p*	<0.001	<0.001	0.33	0.80	012	0.01
Hb (g/dL)	*r*	0.44 **	0.47 **	−0.04	0.30 **	0.32 **	0.21 *
	*p*	<0.001	<0.001	0.74	0.01	<0.001	0.03

BMI = body mass index; ${\overset{\text{˙}}{V}O}_{2\max}$ = maximal oxygen consumption; mL/kg/min = milliliters of oxygen used in one minute per kilogram of body weight; mmol/L = millimoles per litre; μg/L = micrograms per liter; g/dL = grams per deciliter; Hb = hemoglobin; SBJ = standing broad jump * statistical significance set at *p* ≤ 0.05; ** statistical significance set at *p* ≤ 0.01.

**Table 5:** Adjusted for age correlation coefficient *r* between selected physical fitness components and iron status, separately for boys and girls.

		Boys		Girls	
		${\overset{\text{˙}}{V}O}_{2\max}$	SBJ	${\overset{\text{˙}}{V}O}_{2\max}$	SBJ
Serum Iron (mmol/L)	*r*	−0.02	0.22	0.04	0.11
	*p*	0.86	0.08	0.65	0.26
Serum Ferritin (µg/L)	*r*	−0.12	0.005	0.12	0.23
	*p*	0.31	0.96	0.22	0.02
Hb (g/dL)	*r*	−0.05	0.26 *	0.20 *	0.21 *
	*p*	0.70	0.03	0.05	0.03

${\overset{\text{˙}}{V}O}_{2\max}$ = maximal oxygen consumption; mL/kg/min = milliliters of oxygen used in one minute per kilogram of body weight; mmol/L = millimoles per litre; mg/dL = milligrams (mg) per deciliter (dL); μg/L = micrograms per liter; g/dL = grams per deciliter; Hb = hemoglobin; SBJ = standing broad jump * statistical significance set at *p* ≤ 0.05.

## Data Availability

Data for the study are not available as online material but are available from the authors upon reasonable request in accordance with the NWU policy guidelines.
